# Anxiolytic-*like* Effects by *trans*-Ferulic Acid Possibly Occur through GABAergic Interaction Pathways

**DOI:** 10.3390/ph16091271

**Published:** 2023-09-07

**Authors:** Md. Shimul Bhuia, Md. Rokonuzzman, Md. Imran Hossain, Siddique Akber Ansari, Irfan Aamer Ansari, Tawhida Islam, Md. Sakib Al Hasan, Mohammad S. Mubarak, Muhammad Torequl Islam

**Affiliations:** 1 Department of Pharmacy, Bangabandhu Sheikh Mujibur Rahman Science and Technology University, Gopalganj 8100, Bangladesh; shimulbhuia.pharm@gmail.com (M.S.B.); rokonuzzman.phr@gmail.com (M.R.); imranbsmrstuphr@gmail.com (M.I.H.); tawhidislam2021@yahoo.com (T.I.); mdsakibalhasan192412@gmail.com (M.S.A.H.); 2 Department of Pharmaceutical Chemistry, College of Pharmacy, King Saud University, P.O. Box 2457, Riyadh 11451, Saudi Arabia; sansari@ksu.edu.sa; 3 Department of Drug Science and Technology, University of Turin, 10124 Turin, Italy; irfanaamer.ansari@unito.it; 4 Department of Chemistry, The University of Jordan, Amma 11942, Jordan

**Keywords:** anxiolytic effect, *trans*-ferulic acid, GABAergic system, molecular docking, in silico

## Abstract

Numerous previous studies reported that ferulic acid exerts anxiolytic activity. However, the mechanisms have yet to be elucidated. The current study aimed to investigate the anxiolytic effect of *trans*-ferulic acid (TFA), a stereoisomer of ferulic acid, and evaluated its underlying mechanism using in vivo and computational studies. For this, different experimental doses of TFA (25, 50, and 75 mg/kg) were administered orally to *Swiss* albino mice, and various behavioral methods of open field, hole board, swing box, and light–dark tests were carried out. Diazepam (DZP), a positive allosteric modulator of the GABA_A_ receptor, was employed as a positive control at a dose of 2 mg/kg, and distilled water served as a vehicle. Additionally, molecular docking was performed to estimate the binding affinities of the TFA and DZP toward the GABA_A_ receptor subunits of α2 and α3, which are associated with the anxiolytic effect; visualizations of the ligand-receptor interaction were carried out using various computational tools. Our findings indicate that TFA dose-dependently reduces the locomotor activity of the animals in comparison with the controls, calming their behaviors. In addition, TFA exerted the highest binding affinity (−5.8 kcal/mol) to the α2 subunit of the GABA_A_ receptor by forming several hydrogen and hydrophobic bonds. Taken together, our findings suggest that TFA exerts a similar effect to DZP, and the compound exerts moderate anxiolytic activity through the GABAergic interaction pathway. We suggest further clinical studies to develop TFA as a reliable anxiolytic agent.

## 1. Introduction

Depression and anxiety disorders are prevalent conditions in various communities across the globe and in primary healthcare settings. It is common for individuals experiencing depression to reveal symptoms linked to anxiety disorders, and conversely, those with anxiety disorders frequently experience symptoms of depression. These disorders can coexist in an individual meeting the diagnostic criteria for both conditions [1,2]. According to the WHO, it is estimated that 301 million individuals suffered from anxiety disorders in 2019, including 58 million children and teenagers. On the other hand, another report by the WHO demonstrated that about 3.80% of people experienced the effects of depression, including 5.0% among adults and 5.70% among adults over 60 years of age (https://www.who.int/news-room/fact-sheets/detail/depression#:~:text=An%20estimated%203.8%25%20of%20the,among%20women%20than%20among%20men (accessed on 20 April 2023)). Around the world, an estimated 280 million people are affected by depression. In its most severe form, depression can soar to the point of suicide. Currently, at least 700,000 people die each year due to suicide (https://www.who.int/news-room/fact-sheets/detail/mental-disorders, accessed on 20 April 2023).

Anxiety may result from several factors, such as mental condition, physical status, the harmful effects of drugs, leading a stressful life, genetics, brain structure, and function, or a combination of these [3,4,5]. In addition, imbalances in certain neurotransmitters, such as dopamine (DA), serotonin (5-HT), and norepinephrine (NE), have been associated with anxiety and depression [6,7]. In this regard, neurologic patients frequently experience anxiety disorders, such as social phobia, obsessive-compulsive disorder, panic disorder, and generalized anxiety disorder, among others. Unfortunately, these conditions often go unnoticed or are mistakenly considered as the expected reactions to neurologic illnesses. However, if left untreated, anxiety disorders can have a profound effect on the well-being and survival rates of neurologic patients, increasing both morbidity and mortality [8]. In the pathogenesis of anxiety and depression, there are several well-known receptors, such as the gamma-aminobutyric acid (GABA) receptors, N-methyl-D-aspartate (NMDA) receptors, glucocorticoid receptors, alpha-amino-3-hydroxy-5-methyl-4-isoxazole propionic acid (AMPA) receptors, 5-HT receptors, and DA receptors. Similarly, “non-classic” receptors, such as opioid receptors, metabotropic glutamate receptors, and insulin receptors, are also indispensable [9,10,11,12]. Gamma-aminobutyric acid type A (GABA_A_) receptors comprising the α1-subunit (α1 GABA_A_ receptors) have recently been engaged in the sedative activity of benzodiazepines, whereas GABA_A_ receptors comprising α2- and α3-subunits (α2 GABA_A_ and α3 GABA_A_ receptors) have been linked to the anxiolytic activities of benzodiazepines [13,14,15]. In contrast, the remaining α5-subunit receptors are comparatively negligible and not primarily involved in producing anxiolytic (anti-anxiety) or motor effects. Instead, these receptors are thought to have a more specific role in memory processes [15,16,17].

First-generation antidepressants, including monoamine oxidase inhibitors and tricyclic antidepressants, work by enhancing the levels of neurotransmitters like 5-HT and NE. In contrast, benzodiazepines, such as diazepam (DZP), are effective in relieving symptoms of anxiety and depression. This is because benzodiazepines have a wide range of effectiveness and are commonly well-tolerated by individuals [18,19,20,21]. Benzodiazepines are now considered adjunctive or second-line medications, though these medications are most efficient for acute anxiety disorders [19,22]. Due to their low efficacy, dependency, and huge side effects, the development of novel antidepressants is essential for improving their safety, efficacy, and tolerability.

*Trans*-ferulic acid (*trans*-4-hydroxy-3-methoxycinnamic acid) is an organic phytochemical and a stereoisomer of ferulic acid (FA), which is extensively dispensed in nature and is present in various foods in the human diet, such as eggplant, tomato, peanuts, rice, wheat, bananas, and pineapples, among others [23]. Different studies have demonstrated that FA exhibits various pharmacological activities, including antimicrobial [23], antioxidant [24,25], anti-inflammatory [26], antifungal [23], anticancer [27,28], antiallergic [29], hepatoprotective [30], and antidiabetic activities [31], as well as neuroprotective effects including against Alzheimer’s disease [32]. Among the various neuroprotective diseases, FA has beneficial effects against Parkinson’s disease [33], anxiety [34], depression [35], epilepsy [36], insomnia [37], psychosis [38], cerebral ischemia/stroke [39], neuropathic pain [40,41], neurotoxicity [42], dementia [43], and multiple sclerosis [44]. Different studies have shown that FA elicits an effective anxiolytic effect through various receptor interaction pathways, such as NMDA receptors [34], 5-HT_1A_ [34,45], and GABA_A_ [46]. However, no studies have specified the anxiolytic activity of TFA or its underlying mechanisms.

Over the course of three decades, computer-aided drug discovery (CADD) and design methods have played an indispensable role in the development of small molecules with significant therapeutic values. These computational techniques have been instrumental in advancing drug research, thus, facilitating the discovery and design of new and important therapeutics [47]. In this context, conventional methods of drug discovery and development are broadly recognized for their time-consuming nature and high cost-effectiveness. The rising expenses and substantial failure rates associated with this traditional method have underscored the necessity for leveraging CADD methods. CADD offers a promising solution to expedite and optimize the drug discovery process by providing cost-effective alternatives and mitigating the risks associated with traditional approaches [48,49]. Since medications have considerable side effects and possible toxicity, it is vital to assess ADMET (adsorption, distribution, metabolism, excretion, and toxicity) features early in the drug development process. These early screening attempts can improve success rates and shorten screening times for potential medication candidates. This technique provides a more efficient and safer medication development process by proactively detecting and removing compounds with negative ADMET characteristics [50,51,52,53]. To screen a library of chemicals against the target of interest, in particular, the normal job of CADD is to reduce the candidates to a few smaller clusters [54]. Based on the preceding discussion, this study aimed to evaluate the anxiolytic effect of TFA in Swiss albino mice. Additionally, we also performed an in silico study to determine the possible molecular interactions behind the observed effect.

## 2. Results

### 2.1. In Vivo Studies

#### 2.1.1. Open Field Study

**Figure 1:**
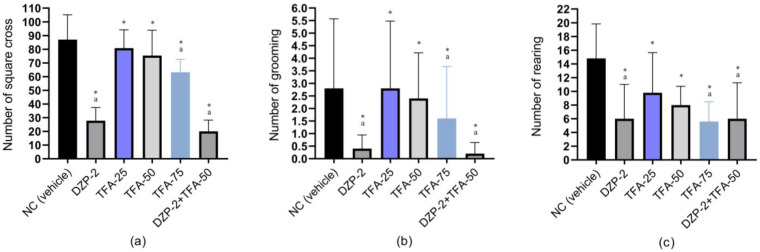
(**a**) Number of square cross (NSC), (**b**) number of grooming (NG), and (**c**) rearing (NR) observed in test and control groups. (Values are the mean ± standard error of the mean (SEM); one-way ANOVA and *t*-Student–Newman–Keuls *post hoc* test with multiple comparisons at 95% confidence intervals; * *p* < 0.05 when compared to the NC (vehicle) group; ^a^ *p* < 0.05 when compared to the TFA-50 group; NC: negative control; DZP: diazepam (2 mg/kg); TFA: *trans*-ferulic acid (25, 50 or 75 mg/kg)).

Results from this investigation showed that pretreated animals in the vehicle group (NC group) exhibit the highest number of square cross (NSC) (87.00 ± 9.08), grooming (NG) (2.80 ± 1.39), and rearing (NR) (14.80 ± 2.51). In contrast, the DZP group (PC group) has a significant (*p* < 0.05) reduction in the NSC (27.80 ± 4.84), NG (0.40 ± 0.27), and NR (6.00 ± 2.50). Results also revealed that the anxiolytic activity of the test sample was dose-dependent; an increase in doses caused a notable decrease in locomotor activity, resulting in a significant (*p* < 0.05) reduction in the NSC, NG, and NR. The mean values for NSC, NG, and NR are 80.80 ± 6.71, 2.80 ± 1.34, and 9.80 ± 2.92, respectively, for TFA-25; 75.40 ± 9.26, 2.40 ± 0.91, and 8.00 ± 1.37, respectively, for TFA-50; 63.20 ± 4.62, 1.60 ± 1.04, and 5.60 ± 1.44, respectively, for the TFA-75 group. In the case of combination therapy, the animals in the combined groups (DZP-2 + TFA-50) exhibited the lowest NSC (20.00 ± 4.12) and NG (0.20 ± 0.22), and an enhanced NR (6.00 ± 2.62) compared to the TFA-75 group and was equal to the DZP group. The NSC, NG, and NR observed in the different treatment groups is displayed graphically in Figure 1.

#### 2.1.2. Hole Cross Study

**Figure 2:**
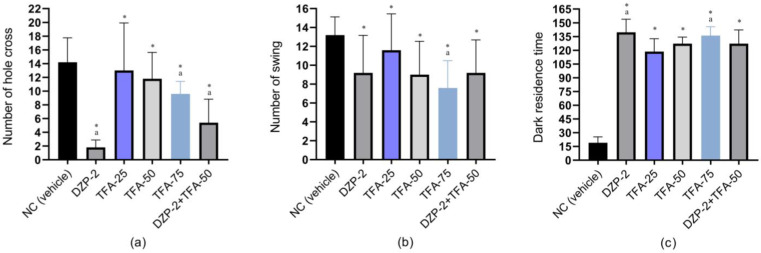
(**a**) Number of holes cross (NHC), (**b**) number of swing (NS), (**c**) dark residence time (DRT) observed in test and/or control groups. (Values are the mean ± standard error of the mean (SEM); one-way ANOVA and t-Student–Newman–Keuls *post hoc* test with multiple comparisons at 95% confidence intervals; * *p* < 0.05 when compared to the NC (vehicle) group; ^a^ *p* < 0.05 when compared to the TFA-50 group; NC: negative control; DZP: diazepam (2 mg/kg); TFA: *trans*-ferulic acid (25, 50 or 75 mg/kg)).

Results from the hole cross test demonstrated that animals in the NC groups show the highest number of hole crosses (NHC) (14.20 ± 1.78), and the NHC significantly (*p* < 0.05) declined in the DZP group (1.80 ± 0.55), exerting a relaxing effect in test animals. Results also showed that the test sample groups (TFA) exhibit a significant (*p* < 0.05) reduction in the NHC compared to the NC groups, in a dose-dependent manner. The values of NHC are 13.00 ± 3.46, 11.80 ± 1.92, and 9.60 ± 0.91 for the TFA-25, TFA-50, and TFA-75 groups, respectively. Moreover, the lowest number of NHC in the combined group was 5.40 ± 1.72. The NHCs of different groups are graphically represented in Figure 2.

#### 2.1.3. Swing Study

Our findings in the swing study showed that the animals in the NC group have the highest number of swings (13.20 ± 0.96), whereas the DZP group exhibited a significant (*p* < 0.05) reduction in the number of swings (NS), compared to the animals in the NC group. The NS for the DZP group is 9.20 ± 1.98. Additionally, our findings showed that animals in different groups of TFA experienced a dose-dependent reduction in the NS, indicating that the lowest NS was demonstrated by the animals in the TFA-75 group (7.60 ± 1.44). In contrast, animals undergoing combination therapy exhibited a lower NS (9.20 ± 1.75) than the NC and lowest dose of TFA (TFA-25), although a higher NS than the other treatment groups in the test sample (Figure 2). The NS for TFA-25 and TFA-50 is 11.60 ± 1.92 and 9.00 ± 0.177, respectively.

#### 2.1.4. Dark–Light Study

In this test, the dark resident time (DRT) was significantly (*p* < 0.05) enhanced in the DZP group compared to the NC group, whereby the animals of these groups resided in the dark box for 19.00 ± 3.25 and 139.60 ± 6.38 s, respectively. Results also indicate that the DRT in different test sample groups significantly (*p* < 0.05) increased dose-dependently compared to the NC groups. The DRT for TFA-25, TFA-50, and TFA-75 is 118.60 ± 7.03, 127.20 ± 3.59, and 136.00 ± 4.83 s, respectively. Furthermore, animals in combined groups (DZP-2 + TFA-50) showed an elevated DRT (127.20 ± 7.54 s) in the dark box compared to the NC group, yet a reduction in resident time was found in the case of DZP administered alone. The DRT of all treatment groups is displayed in Figure 2.

### 2.2. In Silico Study

#### 2.2.1. GABA_A_ Receptor Homology Modeling

**Figure 3:**
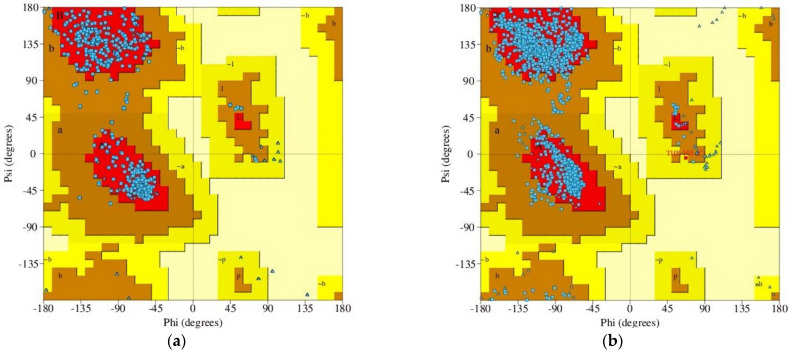
Ramachandran plot of the homology modeled GABA_A_ receptor: (**a**) α2 subunit; (**b**) α3 subunit.

Results of homology modeling indicate that the sequence similarity of the target sequence of GABA_A_ receptor α2 and α3 subunits is 57.18 and 76.50%, respectively, matched with the template sequence. The homology model of the human GABA_A_ receptor was designed with GMQE values of 0.60 and 0.66 for the α2 and α3 subunits, respectively, suggesting good quality and reliability of the designed receptors. The Ramachandran plot was evaluated to validate the precision and dependability of the residues’ Psi and Phi angles. The plot showed 92.70% and 91.90% of the most favored regions of the modeled receptors, as well as 7.30% and 8.0% additionally allowed regions for the GABA_A_ receptor α2 and α3 subunits, respectively. The plot also expressed 0.00% disallowed regions for both receptor subunits (Figure 3).

#### 2.2.2. Molecular Docking and Visualization of Ligand–Receptor Interactions

Molecular docking is performed to estimate the probable binding affinity and interactions between ligands and receptors. In our in silico study, the highest binding affinity (−7 kcal/mol) was expressed by DZP toward the GABA_A_ receptor α3 subunit by forming different types of bonds. We found that DZP revealed −5.9 docking scores against the GABA_A_ receptor α2 subunit. On the other hand, the test ligand TFA revealed the highest interactions and binding affinities toward the GABA_A_ receptor α2 subunit among the two GABA_A_ receptor subunits responsible for the anxiolytic activity. The TFA docking scores are −5.8 and −5.3 kcal/mol for the GABA_A_ receptor subunits α2 and α3, respectively (Table 1).

**Figure 4:**
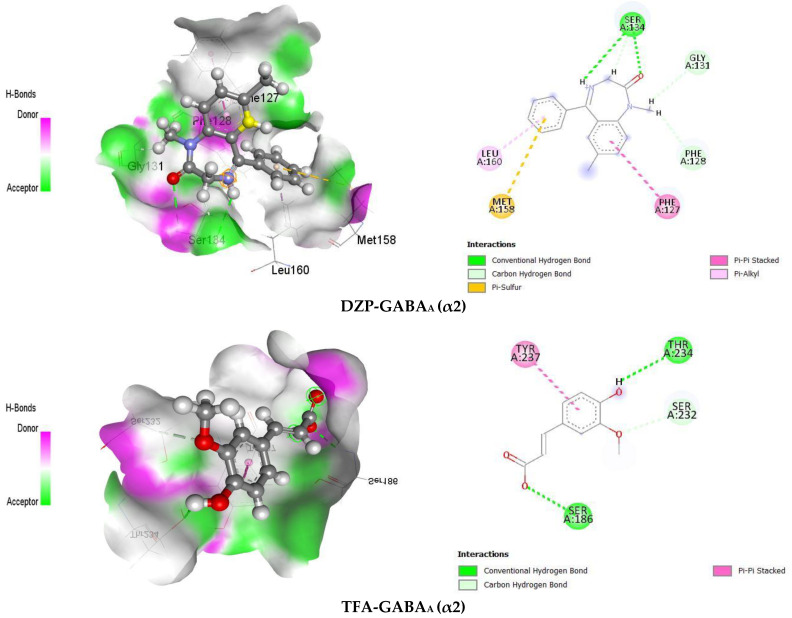

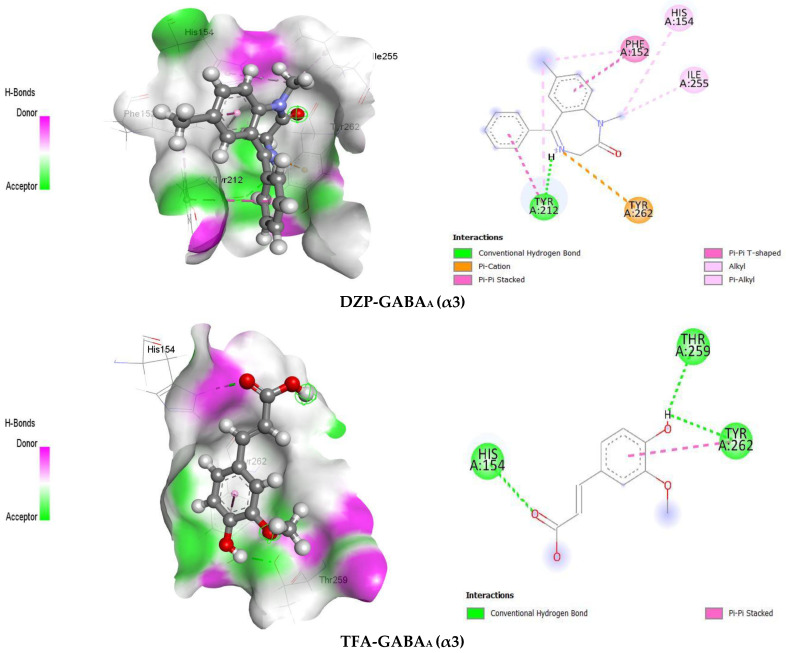
Schematic of 2D and 3D views of the receptor binding site with names of non-bond interactions and interacted amino acid residues between GABA_A_ receptor subunits (α2 and α3) and selected ligands.

To interact with the GABA_A_ receptor, TFA formed various types of bonds, including hydrogen bonds (conventional and carbon–hydrogen bonds) and several types of hydrophobic bonds (alkyl, pi–pi stacked, pi–sulfur, pi–cation, pi–pi T-shaped, and pi–alkyl). TFA interacted with the GABA_A_ (α2) receptor through hydrogen bonds (HB) with the AA residues of SER186, THR234, and SER232 and also formed a pi–pi stack with the AA residue of TYR237. In contrast, the referral drug (DZP) interacts with the GABA_A_ (α2) receptor by making HB with the AA residues of SER134, GLY131, and PHE128, and several other hydrophobic bonds, such as pi–sulfur, pi–pi stack, and pi–alkyl with the AA residues of MET158, PHE127, and LEU160, respectively. Furthermore, our findings showed that TFA forms three HBs with the other subunit (α3) of the GABA_A_ receptor, with the AA residues of HIS154, THR259, and TYR262, and a pi–pi stacked with the AA residues of TYR262. On the other hand, DZP formed a single HB with the AA residue of TYR212 and several hydrophobic bonds, including pi–cation, pi–pi stacked, alkyl, pi–pi T-shaped, and pi–alkyl, with the AA residues of TYR262, TYR212, PHE152, ILE255, and HIS154, respectively. The related AA residues and 2D and 3D visualization involved in the interactions of different kinds of GABA_A_ receptor subunits and ligands are displayed in Table 1 and Figure 4, respectively.

## 3. Discussion

Anxiety disorders are categorized as a cluster of mental conditions characterized by the presence of heightened fear and excessive worry in individuals [55,56]. Anxiety is relevant to the state experienced by an individual when facing a potential predatory attack, whereas fear corresponds to the state of an individual when encountering or being in immediate proximity to a predator [57]. There are various experimental procedures to determine anxiety and develop an efficacious anxiolytic drug [58,59,60]. The majority of current anxiolytic medication discovery focuses on particular biochemical pathways and phenotypic domains. However, using animal models can help to better understand the mechanisms of action [56].

The OFT was designed to evaluate emotionality in animals and is based on exposing an animal to an unfamiliar environment from which it cannot escape due to surrounding walls [61,62]. In this experimental method, anxiety behavior is triggered by the animal’s separation from its social group and agoraphobia [63]. In the OFT, anxiolytic medications decrease animal interest in unfamiliar situations by reducing locomotor activity [64,65]. Locomotor activity is a sign of alertness, and a decline in it implies a drop in CNS excitability [64,66]. Treatment with DPZ reduced the locomotor activity in the anxious test mice [67,68], resulting in calming behaviors [69]. Findings from this investigation showed that treatment of animals with DPZ and TFA significantly reduces locomotor activity, resulting in a diminution in movement and calming behaviors, as demonstrated by the reduction in square crosses, rearing, and grooming. Results also indicated that TFA-treated mice in all groups acquired calming behaviors dose-dependently.

The hole cross test and swing test are widely used experimental procedures for emotional behaviors, such as anxiolytic effects [70,71]. Animals that move normally frequently go through the hole in the boarded box, and in the same way, the movements of the experimental animal inside the swing box cause the box to swing [70,72]. In contrast, animals experiencing a remarkable diminution in the number of movements elucidated as a decrease in inquisitiveness about the new environment, are deemed calming. The significant decline in spontaneous motor activity could be taken as an anxiolytic activity because of the activation of the GABAergic system [71,73]. In our experiment, animals in the test (TFA group) and control groups (DZP) exhibited a lower number of hole crossings and swings in the swing box than those in the vehicle group, resulting in calming behaviors. In this respect, our findings revealed that TFA-treated animals show a dose-dependent response.

The dark–light (DLT) test is a frequently employed mouse test of unconditioned anxiety-like behavior, which is based on a conflict between approach and avoidance between the desire to explore new places and an aversion to clearly lit, open spaces [74]. In the dark–light test, a greater amount of time spent by animals in a particular compartment indicates anxiolytic effects, while the increased movement of animals signifies anxiogenic effects [75]. In this context, previous studies showed that FA reduces the motor activity of mice, resulting in a reduction in movement [76]. In contrast, animals in the vehicle group preferred to reside in lit chambers and spent more time in the light. On the other hand, animals treated with DZP and TFA largely increased their resident time in dark chambers and lowered their movement.

**Figure 5:**
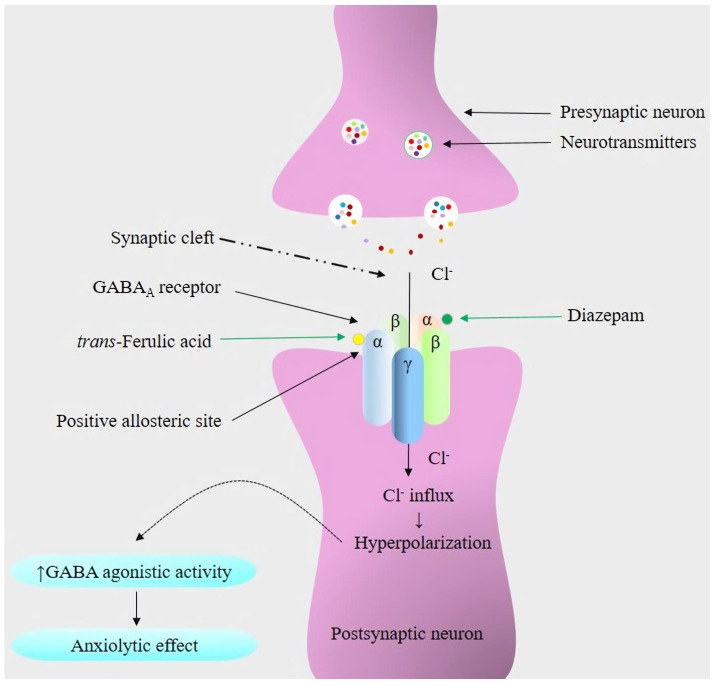
Proposed molecular anxiolytic mechanisms of *trans*-ferulic acid and diazepam with the GABA_A_ receptor. (↑) indicates an increase, and the green arrow shows the binding of the ligands (*trans*-ferulic acid and diazepam) to the positive allosteric sites of different GABA_A_ receptor subunits.

The pathophysiology underlying anxiety disorders is mostly associated with the dysfunction of GABAergic neurotransmission [77]. In our study, we observed that the treatment of animals with DZP decreases locomotor activity, which agrees with results reported by other researchers [67]. DZP and other benzodiazepine drugs act as positive allosteric modulators of the GABA_A_ receptor complex. Although these drugs do not produce an effective response on their own, they enhance the response of the endogenous ligand. These drugs bind to a specific site located at the interface of α and γ subunits in the receptor. The interaction of DZP with these sites leads to an enhanced influx of chloride ions in neurons upon GABA binding. This causes a hyperpolarization in the postsynaptic membranes, thereby enhancing the central nervous system’s depressive response to endogenous GABA [78]. The potentiating effects of GABA are observed in several locations in the brain, including the thalamus, hypothalamus, limbic system, and cerebral cortex. These actions cause a calming effect on neuronal processes within these areas, ultimately, leading to anxiolytic (anti-anxiety) effects [10,79]. Research findings have demonstrated that TFA evokes anxiolytic activity in Zebrafish, possibly by binding at the site of the GABA_A_ receptor, where benzodiazepines exert their effect after binding [46]. Findings from this study reveal that TFA-treated mice exhibit calming behaviors at all experimental doses (25, 50, and 75 mg/kg), where the locomotor activities of the mice were significantly reduced. This indicates the interaction of TFA with the GABAergic system, as these receptors diminished locomotion activity and produced calming behaviors to provide an anxiolytic effect (Figure 5) [80,81].

When two or more medications that have roughly comparable effects are combined, the effects are sometimes considerably increased. The combination is said to be synergistic when the combined effect exceeds that estimated by their potential [82]. Drug synergism usually permits most medication doses to be lowered, thus, lowering the risk of side effects, minimizing the development of resistance, and improving treatment response. Combining drugs with different and distinct mechanisms of action can result in synergistic effects [83,84]. Additionally, synergistic combinations can boost both therapeutic potency and efficacy [85]. Along this line, a different method for increasing the success rate of drug repositioning is to use drug combinations of two or more drugs with diverse mechanisms of action. Therefore, the utilization of combined drug therapy could raise the success rate of finding a new clinical application for a new indication [86]. Our findings from this work revealed that the combined group (DZP + TFA-50) significantly reduced the number of square crosses and grooming but did not change the results in the numbers of rearing and swings compared to the DZP administered alone. In addition, our results indicated that the number of hole crosses was elevated compared to the DZP group but significantly less in comparison to the different groups of TFA. The same result was observed in the DLT, whereby the dark resident time was reduced compared to the DZP group. From the overall findings of the combined group in different tests, it was estimated that the locomotor activity of the test animals was only diminished in the combined group in the cases of hole crossing and grooming tests, compared to the animals in the other groups. In contrast, TFA did not have any synergistic effects when combined with DZP because the maximum experimental findings (NR, NHC, NS, and DRT) suggested a low capability of locomotor activity reduction. In this situation, we can suggest that TFA exerted modulatory effects over DZP but was not synergistic when administered in combination with DZP.

CADD is increasingly significant in the field of drug discovery, playing a crucial role in the cost-effective identification of potential drug candidates. These methodologies facilitate the rational design of novel and safe drugs and the repositioning of existing marketed drugs. They provide valuable support to medicinal chemists and pharmacologists throughout the drug discovery process, aiding in the selection and optimization of promising drug candidates [51,87]. Although molecular docking allows for the discovery of new compounds of therapeutic interest by estimating ligand–target interactions at the molecular level, it also gives the ligand–receptor interaction affinity and estimates the interacting site [88].

In our investigation, TFA expressed the highest binding affinity (−5.8 kcal/mol) against the α2 subunit among the two subunits of the GABA_A_ receptor liable for anxiolytic activity [15], whereas the standard drug DZP showed a binding affinity (−5.9 kcal/mol) of TFA, thereby indicating an almost similar affection toward the receptor. In our view, the α2 subunit of the GABA_A_ receptor contributed more to the potential anxiolytic activity of TFA. Research findings report that HBs have an important impact on the specificity of ligand binding [89]. Results of this work showed that TFA formed 3 HBs with both subunits of the GABA_A_ receptors and several hydrophobic bonds. The ligand–receptor visualization demonstrated that DZP interacts with GABA_A_ (α3) with the AA residues of TYR262 and HIS154, whereas TFA also interacted with this receptor with the same AA residues, thereby indicating the same pocket for the two ligands and proving the capability of TFA for an anxiolytic effect. Therefore, we proposed that TYR262 and HIS154 are the key residues for both ligands in the case of the GABA_A_ (α3) receptor’s anxiolytic activity.

Taken together, the result of our investigation demonstrated that TFA exerts moderate anxiolytic activity since the lead can reduce the locomotor activity of experimental animals dose-dependently, and more specifically, we can propose that the anxiolytic activity of TFA is due to the positive allosteric modulatory effect toward the α2 subunit of GABA_A_ receptors, as the ligand expressed an elevated binding affinity toward this subunit. However, an extensive investigation was needed to check the concentrations or levels of different anxiety-related neurotransmitters, such as DA, NE, and 5HT in the synaptic cleft and postsynaptic neuron, as well as the uptake of the neurotransmitters in the presynaptic neuron, to understand the exact anxiolytic mechanism of TFA.

The major limitation of this study is that the findings may influence various factors, such as the intensity of light or sudden sound from external sources, or from the observer, although we tried to constantly maintain these factors for each animal. Additionally, some animals have the probability of not inducing fear or anxiety through isolation or environmental changes due to their different biological or mental structures. The results may also be affected due to gender discrimination (we used both genders), as scientific evidence demonstrated that the female gender has a higher probability of being anxious. Moreover, it is a comparison study, meaning the findings of this study do not predict the exact anxiolytic mechanisms of TFA; instead, the proposed mechanism is based on the in silico study and on the anxiolytic effect of DZP.

## 4. Materials and Methods

### 4.1. In Vivo Study

#### 4.1.1. Reagents and Chemicals

TFA (*trans*-4-hydroxy-3-methoxycinnamic acid), a 99% mixture of isomers (CAS No.537-98-4) was purchased from Sigma-Aldrich (St. Louis, MO, USA), while the referral drug diazepam (DZP) was obtained from Square Pharma Ltd., Dhaka, Bangladesh.

#### 4.1.2. Preparation of Test and Referral Drugs

We selected three (lower, middle, and higher) concentrations of the test sample based on a literature review. We prepared the mother solution of the test sample at a 75 µg/mL concentration by dissolving it in distilled water (DW). Then, the mother solution was diluted to 50 and 25 µg/mL of the concentration. Additionally, the referral drug (diazepam) solution was prepared by vigorous mixing into DW at concentrations of 2 µg/mL. The middle dose (50 mg/kg) of TFA was selected to be administered in the combination therapy because it demonstrated better activity when administered alone and to avoid excess use of TFA and DZP.

#### 4.1.3. Experimental Animals

Swiss albino mice (22–25 g) of either sex, purchased from the animal house of Jahangirnagar University, Savar, Bangladesh, were used throughout this work. These animals were housed at a constant temperature of 25 ± 1 °C, with regulated lighting (12 h dark/light cycle) at the Pharmacology Lab of Bangabandhu Sheikh Mujibur Rahman Science and Technology University, Gopalganj, Bangladesh, until the experiments started. Animals were given free access to a standard diet and water, and they were kept under standard conditions approved by the Department of Pharmacy at the BSMRSTU (#bsmrstu/phrt16-03). This study also followed the ‘’3Rs alternatives’’ that were described by the Ethical Board. Experiments were conducted from 08:00 a.m. to 3:00 p.m., and animals were monitored for 17 h to check for possible mortality after the study.

#### 4.1.4. Study Design

Animals used in experiments underwent fasting for six hours before the test. Then, a total of 42 animals were randomly divided into 6 groups of 7 animals each. These groups were designated as Gr.-I to Gr.-VI. DW was provided as the negative control, while DZP was administered orally as the positive control. All the doses of TFA were administered orally and the middle dose (50 mg/kg b.w.) of TFA was provided in combination with DZP to investigate the synergistic effects in a separate group of animals. Based on the weight of each mouse, the dosages of the sample substance and the control medications were adjusted. The different treatment groups and their doses are displayed in Table 2.

##### Open Field Test (OFT)

The open field apparatus consisted of a wooden open field area with a pointed square floor (30 × 30 × 30 cm^3^). The number of square crossing (NSC), grooming (NG), and rearing (NR) were counted manually over 5 min. After recording these parameters for each animal, the ground of the experimental apparatus was cleaned with 70% ethanol [90].

##### Hole Cross Test (HCT)

In this experiment, we used a wooden apparatus of 30 × 20 × 14 cm^3^. A 3 cm diameter hole was drilled just 1 inch above the lowest part of the dividing board in the cage. After 3 min of the previous study (OFT), each animal was placed on one end of the apparatus. The mice were visible, using the hole to freely move between rooms for five minutes. The number of holes was counted manually. The floor of the apparatus was also thoroughly cleaned, as mentioned above [91].

##### Swing Test (ST)

The swing test apparatus consisted of a 120 g PP (polypropylene) swing box (21.5 × 12.5 × 11.5 cm^3^) set on a fixed rod (42.5 × 1.5 cm^2^). The infrastructure made up the entire setup, which measured 36.5 × 29 × 2 cm^3^. Wood was used to construct the stage and supports, while stainless steel (SS) was employed as a swing rod. This SS rod was bisectionally (equally) installed on the lower part of the swing box. Three minutes after the previous study (HCT), each animal was placed on one end of the apparatus. The number of swings (NS) for each mouse was counted manually for five minutes. The floor of the PP swing box was also thoroughly cleaned, as mentioned above [92].

##### Dark–Light Test (DLT)

The study apparatus was made of wood and divided into two chambers, the lightbox, and the dark box, which was joined by a little door. The lightbox (27 × 18 × 29 cm^3^) was brighter than the dark one (black portion: 27 × 18 × 29 cm^3^) and was illuminated by ambient light. Three minutes after the previous study (ST), each animal was kept in the light portion of the apparatus. The time spent in the dark box was counted manually using a stopwatch for three minutes. The floor of the apparatus was thoroughly cleaned, as mentioned above [93].

#### 4.1.5. Statistical Analysis

Results are expressed as the mean ± standard error of the mean (SEM). Data were subjected to one-way analysis of variance (ANOVA followed by *t*-Student–Newman–Keuls *post hoc* test using the statistical software GraphPad Prism (version 9.5) (GraphPad Software, San Diego, CA, USA, http://www.graphpad.com (accessed on 20 April 2023)), and experimental groups were compared against the vehicle (control) group; differences were considered significant at *p* ≤ 0.05 at 95% confidence intervals.

### 4.2. In Silico Study

#### 4.2.1. Homology Modeling and Preparation of Receptors

Based on the existing literature, we selected two subunits (α2 and α3) of the GABA_A_ receptor liable for the anxiolytic activity to conduct molecular docking and ligand–receptor visualization [13,14,15]. Due to the unavailability of the 3D structures for the selected GABA_A_ receptor subunits in the RCSB Protein Data Bank [94], we developed a homology model to obtain the required 3D structures. The SWISS-MODEL was utilized to perform the homology model and obtain the desired receptors [95]. The sequences of the receptor subunits α2 (UniProt ID: P47869) and α3 ((UniProt ID: P34903) were collected from the UniProt database (http://www.uniprot.org/ (accessed on 18 May 2023)) [96], then, a BLAST assessment was performed with the aid of the NCBI BLAST [97] tool to choose the template. The GABA_A_ homology modeling structures were assessed by GMQE [98] and a Ramachandran plot, via ProCheck [99,100,101]. After collection and developing homology, the receptors were fully optimized to eliminate docking interference by removing all unimportant molecules, and macromolecules, such as lipids, heteroatoms, and water molecules from the sequence of designated receptors using the PyMol software package (v2.4.1) [102,103,104]. Eventually, the receptors underwent energy minimization and geometry optimization using the SwissPDB Viewer software package. This process used the GROMOS96 force field, and the resulting PDB file was saved for subsequent molecular docking analysis.

#### 4.2.2. Collection and Preparation of Ligands

**Figure 6:**
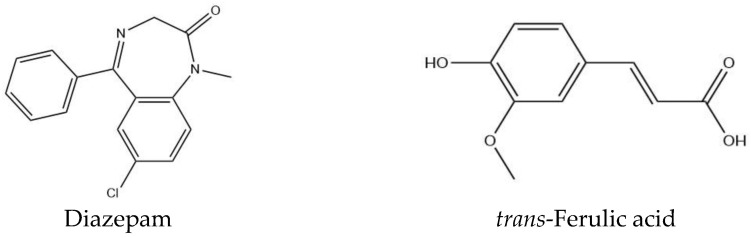
Chemical structures of standard and test compounds.

The 3D conformers of diazepam (Compound CID: 3016) and *trans*-ferulic acid (Compound CID: 445858) were downloaded in SDF format from the PubChem chemical database (https://pubchem.ncbi.nlm.nih.gov/ (accessed on 18 May 2023)). Subsequently, the 3D conformers of the selected ligands underwent energy minimization via the Chem3D 16.0 program package. Then, the minimized conformers were saved as SDF files in preparation for the molecular docking process. In this respect, Gaussian View software (v5.0) was employed to optimize all the ligands. The 2D chemical structures of the ligands are depicted in Figure 6.

#### 4.2.3. Molecular Docking and Visualization of Ligand–Receptor Interactions

Molecular docking to estimate the active binding affinity of the selected ligands against the active sites of GABA_A_ receptors was conducted with the aid of the PyRx software package. To carry out molecular docking, the dimensions of the gird box were set as 80 × 80 × 80 Å along the x-, y- and z-axes, respectively, and the calculation was run at 200 steps [105]. The outcome of the docking potential is stored in ‘.csv’ format, while the ligand–protein complex is saved in PDB format. Additionally, the ligand is collected in PDBQT format for further analysis. We used the Discovery Studio Visualizer (v21.1.020298) and PyMol (v2.4.1) software applications to determine the interactions between ligands and receptors and the receptor’s active site. Table 1 lists the interacting amino acid (AA) residues and bond types between the ligand–receptor interactions.

## 5. Conclusions

In summary, the findings from this investigation demonstrated that TFA displays significant anxiolytic activity, as the compound reduces locomotor activity in experimental animals and induces calming behaviors. In addition, computational investigations revealed that TFA has an elevated binding affinity (−5.8 kcal/mol) toward the α2 subunit of the GABA_A_ receptor among the two subunits (α2 and α3) liable for anxiolytic activity. Our findings also indicated that TFA exerted modulatory effects over DZP but was not synergistic when administered in combination with DZP, which is due to the modulation capability of the allosteric activity of DZP. Taken together, TFA reduced locomotor activity, giving a calming effect to prevent anxiety in experimental animals, possibly through binding with the GABA_A_ receptor. These findings may explain the medicinal use of TFA as an anxiolytic agent. However, more investigation and clinical studies, possibly using human subjects, are required to establish the safety and efficacy of TFA as a reliable anxiolytic agent.

## Figures and Tables

**Table 1:** Docking scores and amino acid residues of ligand–receptors interaction.

Ligands	Receptors	Docking Scores (kcal/mol)	Amino Acid Residues	
			HB	Others
DZP	GABA_A_ (α2)	−5.9	SER134, GLY131, PHE128	MET158, PHE127, LEU160
TFA	GABA_A_ (α2)	−5.8	SER186, THR234, SER232	TYR237
DZP	GABA_A_ (α3)	−7.0	TYR212	TYR262, TYR212, PHE152, ILE255, HIS154
TFA	GABA_A_ (α3)	−5.3	HIS154, THR259, TYR262	TYR262

DZP: diazepam; TFA: *trans*-ferulic acid; HB: hydrogen bond.

**Table 2:** Groups and treatments via oral administration.

Treatment Groups	Description (R/A)	Dose (mg/kg)
Gr.-I: NC	Vehicle: Distilled water (p.o)	10
Gr.-II: DZP	Standard: Diazepam (agonist) (p.o)	2
Gr.-III: TFA	Lower dose (p.o)	25
Gr.-IV: TFA	Medium dose (p.o)	50
Gr.-V: TFA	Higher dose (p.o)	75
Gr.-VI: TFA + DZP	Test + Standard combination (p.o)	50 + 2

## Data Availability

Data is contained within the article.
